# A Mobile Health Intervention to Support Collaborative Decision-Making in Mental Health Care: Development and Usability

**DOI:** 10.2196/57614

**Published:** 2025-01-17

**Authors:** Kristin Lie Romm, Mari Skoge, Elizabeth Ann Barrett, Lars-Christian Berentzen, Dagfinn Bergsager, Pål Fugelli, Thomas Bjella, Erlend Strand Gardsjord, Kristine Kling, Sindre Hembre Kruse, Kari Jorunn Kværner, Ingrid Melle, Erlend Mork, Henrik Myhre Ihler, Eline Borger Rognli, Carmen Simonsen, Tor Gunnar Værnes, Sofie Ragnhild Aminoff

**Affiliations:** 1 Early Intervention in Psychosis Advisory Unit for South-East Norway Division of Mental Health and Addiction Oslo University Hospital Oslo Norway; 2 Institute of Clinical Medicine University of Oslo Oslo Norway; 3 Section for Early Intervention in Psychosis Division of Mental Health and Addiction Oslo University Hospital Oslo Norway; 4 University Center for Information Technology University of Oslo Oslo Norway; 5 Department of Research and Innovation Division of Mental Health and Addiction Oslo University Hospital Oslo Norway; 6 Centre for Connected Care (C3) Oslo University Hospital Oslo Norway; 7 BI Norwegian Business School Oslo Norway; 8 Section for Clinical Psychosis Research Division of Mental Health and Addiction Oslo University Hospital Oslo Norway; 9 Nydalen DPS Division of Mental Health and Addiction Oslo University Hospital Oslo Norway; 10 Section for Clinical Addiction Research Division of Mental Health and Addiction Oslo University Hospital Oslo Norway

**Keywords:** eHealth, shared decision-making, user involvement, user-centered design, mental disorder, mobile technology, illness course, recovery, mobile apps, mHealth

## Abstract

**Background:**

Shared decision-making between clinicians and service users is crucial in mental health care. One significant barrier to achieving this goal is the lack of user-centered services. Integrating digital tools into mental health services holds promise for addressing some of these challenges. However, the implementation of digital tools, such as mobile apps, remains limited, and attrition rates for mental health apps are typically high. Design thinking can support the development of tools tailored to the needs of service users and clinicians.

**Objective:**

This study aims to develop and beta test a digital tool designed for individuals with severe mental disorders or substance use disorders to facilitate shared decision-making on treatment goals and strategies within mental health services.

**Methods:**

We used a user-centered design approach to develop iTandem, an app facilitating collaborative treatment between service users and clinicians. Through qualitative interviews and workshops, we engaged 6 service users with severe mental disorders or substance use disorders, 6 clinicians, and 1 relative to identify and design relevant app modules. A beta test of iTandem was conducted to refine the app and plan for a pilot trial in a clinical setting. After 6 weeks of app use, 5 clinicians and 4 service users were interviewed to provide feedback on the concept, implementation, and technical issues. Safety and ethical considerations were thoroughly discussed and addressed.

**Results:**

To avoid overload for the service users, we applied a pragmatic take on module content and size. Thus, iTandem includes the following 8 modules, primarily based on the needs of service users and clinicians: Sleep (sleep diary), Medication (intake and side effects), Recovery (measures, including well-being and personal recovery, and exercises, including good things and personal strengths), Mood (mood diary and report of daily feelings), Psychosis (level of positive symptoms and their consequences and level of negative symptoms), Activity (goal setting and progress), Substance use (weekly use, potential triggers or strategies used to abstain), and Feedback on therapy (of individual sessions and overall rating of the past week). For the beta testing, service users and clinicians collaborated in choosing 2-3 modules in iTandem to work with during treatment sessions. The testing showed that the app was well received by service users, and that facilitation for implementation is crucial.

**Conclusions:**

iTandem and similar apps have the potential to enhance treatment outcomes by facilitating shared decision-making and tailoring treatment to the needs of service users. However, successful implementation requires thorough testing, iterative development, and evaluations of both utility and treatment effects. There is a critical need to focus on how technology integrates into clinical settings—from development to implementation—and to conduct further research on early health technology assessments to guide these processes.

## Introduction

...in what way has it (iTandem) been helpful?

Like right now, I just went into the “substance use” module to write that I managed to say ‘no’ to buying heroin. Or if I see needles and stuff, I choose to try and get away – move away from where there are needles and that kind of stuff available.

Beta tester of iTandem

The ability to communicate effectively with service users is essential in health care. Therapeutic communication can lead to several benefits, including a more accurate description of symptoms, improved detection of emotional states, more appropriate therapeutic measures, better identification of the service user’s needs and understanding of their disorder and treatment, and an increased likelihood of shared decision-making between clinicians and service users [1-3]. Engaging individuals with severe mental disorders, substance use disorders, or both in their recovery process can be challenging. Various factors contribute to this difficulty, including a lack of trust in health care services [4], poor therapeutic alliance [5], reduced insight into their illness [6], diminished cognitive functioning [7], and issues with motivation and apathy [8,9].

Priebe and colleagues [10] outlined 5 guiding principles for effective communication in mental health services: focusing on the service users’ concerns, demonstrating positive regard and personal respect, appropriately involving service users in decision-making, showing genuineness with a personal touch, and using a psychological treatment model. Emphasizing service users’ concerns aligns closely with the concept of “patient-centeredness” in health care. A recent review [11] defined centeredness as encompassing attributes such as being treated as unique, being heard, and sharing responsibility. However, Feldthusen and colleagues [11] emphasize that centeredness is a complex concept, with its essence rooted in the interactions and communication between clinicians and service users. For individuals with severe mental illness, achieving a sense of positive regard and personal respect can be challenging. Therefore, adopting a nonjudgmental approach has been shown to be particularly valuable [12]. Shared decision-making is a widely recognized concept in medicine, but it remains challenging to implement effectively in mental health care. For individuals with severe mental disorders, barriers include clinicians’ paternalistic approaches to care [13] and service users’ internalized stigma [14]. Increasing service user involvement in activities such as goal setting, addressing values and preferences, and working with motivational factors is essential [15]. Additionally, exploring shared risk-taking can further enhance communication [16]. Genuineness and a personal touch involve openness and sharing but also reflect the therapist’s behavior and characteristics, such as warmth and flexibility [17]. Finally, adopting a coherent client-centered therapy approach, such as solution-focused therapy, problem-solving therapy, or cognitive behavioral therapy, is often preferable [10].

Digitalization offers a potential solution to some of these challenges by making treatment more focused and efficient. There has been growing interest in the use of patient-reported outcomes and patient-reported outcome measures (PROMs) in health care [18], which are frequently digitalized. The inclusion of validated assessment tools in digital formats provides clear advantages. However, a significant barrier for health professionals is the time and expertise required to interpret and integrate the extensive findings into their workflow [19]. Additionally, Nguyen and colleagues [19] identified perceived irrelevance and lack of value for the service user as barriers to completing PROMs. As a result, both service users and clinicians emphasize the need for tools that are functional, straightforward, and concise, with as few questions as possible.

There has been a surge in smartphone apps designed to support individuals with mental health challenges, readily available on platforms such as the App Store (Apple Inc.) or Google Play (Alphabet Inc.). Most of these apps are standalone offerings in the free market, focusing on symptoms such as depression, anxiety, or stress. However, they are rarely developed to meet the safety standards typically required in health care settings, making integration into clinical practice difficult. Additionally, while many apps appear acceptable and feasible initially, attrition rates over time are often high [20]. Individuals with mental disorders may face unique challenges that demand flexible solutions, as therapeutic targets can shift throughout the course of illness. Thus, assessing attrition alone is often insufficient without a deeper understanding of the context. Proposed solutions include placing greater emphasis on external motivating factors and user experience (UX) design, as well as integrating digital mental health solutions into existing care pathways [21,22].

KISS—“keep it simple, stupid”—is a design principle [23] frequently used in design processes and should be applied to service design as well [24]. This principle emphasizes that most systems function optimally when kept simple rather than overly complex [25]. Simpler innovations are more likely to be successfully deployed. Aligning with this concept, human-centered design has gained significant attention in recent years. It involves creative approaches that enable end users to influence the design of technology, tailoring it to better meet their needs [26]. One such approach is participatory design. A fundamental principle in this approach is that people affected by a decision should have the opportunity to influence it, with the goal of designing alternatives to improve quality of life [27]. Despite extensive research on the importance of applying design principles to technology to make it effective, only a limited number of studies in mental health care report using co-design and expert designers in their developmental process [26,28].

A previous service design project focused on the establishment of a low-threshold service for people with psychosis in health care services [29]. Based on insights from service users, caregivers, and clinicians, easy access to a first assessment of psychosis was established. During this project, it became evident that individuals with lived experience were interested in new ways of collaborating with health care services. They sought tools that could enhance the effects of treatment, make better use of the time between sessions, and help them stay focused on their goals. As one service user remarked, “I live my life between the appointments.” This highlighted the need to explore how technology could strengthen the focus on treatment between appointments.

The main aim of the iTandem development was to investigate the needs described by service users and clinicians and to integrate them into an app designed to enhance shared decision-making for people with psychotic, bipolar, or substance use disorders in treatment. We applied design thinking principles to incorporate user needs and feedback throughout the process [30,31]. Our long-term goal is to explore the barriers and enablers for integrating technology into shared decision-making in mental health care and to examine how the integration of digital tools affects the mode of service delivery. In this paper, we will present (1) the design process behind the development of iTandem and (2) the results of beta testing. Finally, we will discuss the importance of future research on the digital enhancement of service delivery.

## Methods

### Project Setting

iTandem was developed at the Early Intervention in Psychosis Advisory Unit for South East Norway (TIPS SE) at Oslo University Hospital (OUH). TIPS SE works to support mental health services in detecting and treating patients with early psychosis in accordance with national guidelines, with a particular emphasis on how these services are organized.

### Design Process

#### Overview

External funding from the South-Eastern Norway Regional Health Authority enabled the hiring of experts in service design and interaction design to lead the process. The process progressed through 3 phases, involving multiple stakeholders including patients, clinicians, developers, data protection officers, and clinicians, in addition to the original project team consisting of researchers and clinicians from OUH (KLR, EAB, KK, EM, EBR, CS, TGV, and SRA). The phases involved are discussed in the following subsections.

#### Preparation Phase

Qualitative interviews were conducted to inform the design process by understanding the needs and preferences regarding app content. Six patients diagnosed with psychotic or bipolar disorder, as well as 1 relative, were interviewed individually. In addition, 2 focus groups were held with 6 clinicians: 1 nurse practitioner, 1 clinical psychologist, 1 psychiatrist, and 1 medical doctor (all from an early intervention in a psychosis team); as well as 1 psychologist from a general psychiatric outpatient service and 1 from the child and adolescent services for early psychosis.

#### Insight Phase

A private design company managed the design process and developed a clickable app prototype to inform the developers. The design company, in collaboration with researchers, agreed upon a project plan that included several meetings with clinicians (the same clinicians who had been involved in phase 1) and 4 patients (the same as in phase 1). The designers utilized the insights collected during phase 1 and knowledge generated through workshops to focus on themes that appeared to be both significant and feasible to work with, given the project’s budget and time constraints.

#### Developmental Phase

This phase was carried out in collaboration with the Mobile App team at the Center for Information Technology (USIT) at the University of Oslo (UiO), which developed a working app capable of transferring encrypted data to secure storage within Services for Sensitive Data (Tjenester for Sensitive Data or TSD). This was necessary to meet the safety requirements for the storage of sensitive data, as mandated by the data protection officer at OUH. A risk and vulnerability analysis was conducted at OUH. During this phase, 4 patients and 2 clinicians were invited to test the iTandem app during development to ensure it could be adjusted based on short iterations.

### Project Organization

#### Overview of the Project Group

The project group included researchers and clinicians working with early psychosis treatment. TIPS SE also has a board composed of people with lived experience of bipolar and psychotic disorders. These individuals were either currently in treatment or had previously been in treatment at mental health services in OUH during the development project. The roles of the different groups are presented below.

#### Researchers

During phase 1, researchers conducted individual qualitative interviews with 6 service users and 1 relative and led 1 focus group with 6 clinicians. The purpose was to explore the challenges participants believed digital tools could address. During phase 2, the researchers served as a bridge between the clinic and the design team, facilitating workshops, collaborating with user representatives, and ensuring ethical issues were handled appropriately. They also suggested improvements to the app content to ensure it aligned with clinical guidelines and recommendations regarding treatment. During phase 3, the researchers facilitated and oversaw the developmental process of the app at USIT.

#### Designers

During phase 2, designers planned and conducted workshops with clinicians and researchers. Throughout the project, they interviewed clinicians and user representatives, and user tested different options in the studio with both clinicians and user representatives in an iterative process. The process was based on design thinking, working with divergent and convergent thinking according to the Double Diamond framework [32]. During phase 3, UX designers, engaged in making products or services usable, enjoyable, and accessible, were involved at USIT to further develop the interface and add functionality based on tests from the prototyping phase.

#### Service Users

Service users with severe mental disorders or substance use disorders were involved throughout all phases of the project. Service users (aged 18-30 years) and 1 relative (parent) provided input on functionality and were invited to user test different prototypes. This involvement had an impact on the final app interface and functionality. Some of these service users were among those interviewed during phase 1. Additionally, we met with the board of user representatives in the Department of Substance Use and Addiction at OUH to discuss app content relevant to them. Service users not directly involved in development were invited to beta test iTandem.

#### Clinicians

During the process, involved clinicians highlighted important aspects essential for implementation, and shared their ideas on how iTandem could be valuable in their daily clinical work. Additionally, they user tested the app during iterative cycles and provided feedback on how they believed the app would be received by their patients.

### Software

The iTandem app was developed using the React Native Open-Source UI Software Framework (Meta Platforms, Inc.) and is available for both iOS and Android platforms. It can be downloaded from the App Store or Google Play. Data are transferred via HTTPS as JSON files through a secure web survey provider (Nettskjema), hosted by USIT at the UiO. The “Activity,” “List of Medication,” and “Diary of Good Things” modules are stored on the user’s phone for easy access, while aggregated data from all modules are encrypted and transferred to a secure server (TSD) at UiO once a module is completed. These data generate a report that can be accessed by clinicians during consultations and provide structured data available for researchers.

### Ethical Considerations

The study was approved by the Data Protection Officer at OUH (approval numbers 2016/14408 and 20/18434). The regional ethical committee found that the development and testing fell outside their mandate (reference number 105851). All clinicians and service users who were interviewed signed informed consent forms. Service users who were engaged during the early phases of development, in collaboration with the service designers, also signed informed consent for their involvement in this work. All data were handled securely at TSD and by a secure research server at OUH. Service users were compensated with approximately US $23 per hour. All data used in this paper have been anonymized.

## Results

### The Development Process

#### Designing iTandem for Clinical Integration and Shared Decision-Making

We aimed to make the use of iTandem as true to a clinical setting as possible, keeping both the service user’s and clinicians’ experiences in mind when framing the app content. Our primary goal was to develop a tool to enhance shared decision-making. Besides supporting collaboration, we believe this is crucial for compliance. iTandem is not a standalone tool, and both its usage and the reasons for any attrition should be explored during treatment. We used phrases that closely mirror those used by clinicians. Furthermore, we chose to incorporate only a minimum of validated self-report measures, as the rationale behind the app was to serve as a communicative tool rather than a strict research assessment instrument.

The described needs were in line with current research, and based on the current literature, the rationale behind each module is outlined below (Table 1).

**Table 1:** Description of the modules of iTandem and their intended purposes.

Module	Frequency of notifications	Aim	Contents	Basis of measure/intervention
Sleep	Twice daily	Map sleep patterns and quality, link to the level of function	Sleep diary	Consensus sleep diary
Medication	Once or twice daily	Support service users in keeping track of and remembering to take medication at the right time	Registration of medications taken and potential side effects List of medications	Partly developed by the project group and partly adapted from the UKU Side Effect Scale Developed by the project group
Recovery	Daily/weekly Monthly	Do positive psychology exercises Map and focus on personal recovery and positive aspects of mental health	Good things Personal strengths Psychological well-being Emotional well-being Cognitive well-being	Exercises adapted from “Positive psychotherapy for psychosis” Questionnaire about the Process of Recovery Warrick-Edinburgh Mental Well-Being Scale Lehman Life Satisfaction Scale
Mood	Daily	Map feelings and mood variability, link to sleep, medication, experiences, and other symptoms	Mood diary Emotions	Adapted from the paper version of the mood diary from the Systematic Treatment Enhancement Program for Bipolar Disorder study Developed by the project group
Psychosis	Weekly Monthly	Follow symptom development and relation to difficulties in reaching goals, link to other variables, guide treatment	Symptoms of psychosis Negative symptoms	Developed by the project group Self-evaluation of negative symptoms (Self-Evaluation of Negative Symptoms Scale)
Activity	Daily	Increase/regulate activity level, set goals, plan activities, motivate to complete, bring personal experiences into treatment	Registration of goals Registration of activity	Developed by the project group
Substance use	Weekly	Gain a better understanding of how substance use impacts symptoms and daily life, guide coping strategies	Registration of substance use and coping strategies	Developed by the project group
Feedback on treatment	Before and after consultations	Facilitate feedback to the clinician to strengthen patient involvement and quality of treatment, provide an overview of the treatment course	Before consultation After consultation	Developed by the project group

#### Sleep

Sleep disturbances are common in psychotic and bipolar disorders [33]. In bipolar disorder, sleep disturbances are frequently observed during and between episodes and are associated with more severe symptoms [34]. Therefore, being able to detect and monitor disturbed sleep patterns and implement treatments that focus on improving sleep may be crucial. Both service users and clinicians considered it valuable to have a tool that collects daily data on sleep to avoid recall bias. Additionally, clinicians expressed a need for a tool to assist in diagnosing sleep disorders. The Sleep module was based on the consensus sleep diary, which enables the monitoring of subjective sleep measures and the diagnosing of common sleep disorders [35]. The diary consists of a questionnaire in the morning about how well you slept and a questionnaire in the evening where users rate how their day was. The intended purpose of the Sleep module is to provide an overview of service users’ sleep patterns and, based on this, initiate relevant sleep treatment if necessary. Additionally, the module includes a link to a website [36] with information about common sleep problems in severe mental disorders and sleep hygiene advice.

#### Medication

Both service users and clinicians wanted a tool that could assist with reminders (notifications) for taking medication and monitoring medication side effects. Medication is a key component in the treatment of both psychotic and bipolar disorders. Antipsychotics are effective, especially during the early and acute phase of psychosis [37], and are also commonly used in people with bipolar disorder [38], along with mood stabilizers [39]. However, these medications may have side effects that are not always properly addressed [40]. Recent studies have highlighted the importance of considering the user’s experience level, autonomy processes, values and risk preferences, knowledge, and needs to accurately understand the service users’ experience with medication [41]. The module was designed to enable service users to create a (revisable) list of their medications, set reminders for doses, and report side effects. Previous research has demonstrated that the language used to describe side effects can influence users’ expectations and may contribute to experiencing nocebo-induced side effects [42]. Webster and colleagues [42] found this to be particularly significant for people with negative beliefs about side effects. To mitigate this, we used UX design to integrate the patient-rated UKU Side Effect Scale [43], which consists of 48 single symptom items, into the Medication module. Instead of requiring the service user to rate 48 individual items, we chose to present the scale as a visualization of the human body. Service users can click on the body part where they experience side effects and select the relevant side effect(s).

#### Recovery

Clinicians were primarily interested in how treatment impacted clinical recovery in terms of symptom remission and adequate functioning. However, they also emphasized the importance of considering the impact on quality of life. Service users, by contrast, highlighted the need to focus more on their positive outcomes in line with personal recovery. This is commonly defined as the subjective experience of connectedness, hope, identity, meaning in life, and empowerment [44]. Personal recovery has received increased attention in mental health research in recent years, particularly in relation to psychotic disorders [45,46]. This highlights the importance of monitoring treatment outcomes in terms of well-being, personal recovery, and clinical recovery. Positive psychology has been applied to the treatment of mental disorders and, more recently, to psychotic disorders through positive psychotherapy for psychosis [47]. The Recovery module has 2 parts: First, it monitors personal recovery using a validated assessment measure [48], focusing on good feelings and thoughts with a validated measure of mental well-being (Warrick-Edinburgh Mental Well-Being Scale) [49], and life satisfaction with The Life Satisfaction Scale [50]. Second, the module includes 2 positive psychology exercises, namely, (1) Good Things, which is a positive diary of good experiences. The diary allows for daily notes to be stored on the phone, which can be revisited by the service user. It is based on positive psychology, suggesting that gratitude interventions can positively affect well-being, happiness, and life satisfaction [51]. (2) Personal Strength Exercise, which emphasizes the need for strength-based interventions for people with severe mental disorders [52]. The service user is presented with 24 strengths visualized by icons. They are encouraged to select 4 of their personal strengths and plan activities that utilize these strengths. This approach is adapted from the Positive Psychotherapy for Psychosis group intervention developed by Slade et al [47], in accordance with the WELLFOCUS study [53].

#### Mood

Detecting fluctuations in mood symptoms, such as elevated and depressed moods, is crucial in the treatment and relapse prevention of bipolar disorder, as emphasized by the service users. However, mood symptoms are not limited to bipolar disorder; they also play a significant role in other mental disorders, as highlighted by all the service users and clinicians who were interviewed [54,55]. Monitoring mood and affective symptoms is also relevant for personal recovery [56]. The service users wanted the opportunity to log affective states or emotions as they emerge in daily life, with the option of linking such experiences to external triggers. Service users with bipolar disorder may benefit from using daily mood charts to monitor their symptoms [57]. This feature was incorporated into the Mood module based on a paper version adapted from the Systematic Treatment Enhancement Program for Bipolar Disorder [58]. Additionally, we developed a module to monitor daily emotions, potential triggers, and an evaluation of fluctuating or stable emotions.

#### Psychosis

The clinicians were interested in measures to detect how psychotic symptoms fluctuate and also the ability to detect triggers. This functionality was less important to the service users. This might seem paradoxical but aligns with the research literature on recovery [59]. However, most service users agreed that it could be relevant to log their symptoms to register the effects of medication, although they argued that the questions needed to be brief and relevant. As we defined iTandem as a tool aimed at facilitating dialogue in treatment sessions and not for research assessment, we chose to include questions about psychosis as typically phrased by clinicians in clinical practice. Thus, the Psychosis module includes a list of positive psychosis symptoms to choose from and brief follow-up questions regarding how symptoms affect daily function, triggers, etc. This may serve as a means to monitor treatment progression regarding positive psychotic symptoms. Additionally, the clinicians wanted to monitor negative symptoms. Negative symptoms are challenging to assess due to their overlapping features with, for example, depression and medication side effects [60]. However, the Self-Evaluation of Negative Symptoms Scale has been shown to be a valid self-report scale, and we chose to incorporate this into the Psychosis module [61].

#### Activity

Increasing goal-directed activity is important in the recovery process for severe mental disorders. However, achieving this in treatment can be challenging. The service users and clinicians wanted functionality that could be incorporated as part of treatment and motivate for change. This aligns with previous research, which states that goal planning is an important element in mental health service delivery, but we lack standardized approaches to support it [62]. Furthermore, both working alliance and goal achievement support the individual recovery process, and a strong working alliance seems to make people more capable of reaching their goals [63]. Potentially, digital tools could supplement and enhance this alliance. This module aims to support motivation for activity by allowing the service user to set long- and short-term goals. When an activity that supports the goal has been executed, the service user selects an icon representing this goal. Each completed activity creates a streak.

#### Substance Use

Substance use and abuse are of major concern in both psychotic [64] and bipolar disorders [65]. In addition, people with substance use disorder have a higher prevalence of mental disorders than the general population [66]. However, even though both service users and clinicians agree that this is a major problem, it became apparent that the service users did not want to focus on detailed intake of substances. They stated that thorough monitoring of their weekly substance use did not motivate change and argued for brief questioning on drug intake. However, they found it useful to be able to convey which strategies they had used to abstain from taking drugs during the week. Clinicians were more prone to ask for detailed accounts of substance use to get an overview but were also concerned that this could lead to demotivation and disengagement. In addition, we were guided by principles from motivational interviewing and aimed to engage the service user in strategies to abstain from using drugs when feeling a craving [67]. Hence, we chose the same strategy as with the Psychosis module and included only brief questions about use and abstaining strategies, along with a link to a recommended user-friendly web page about substance use.

#### Treatment

**Figure 1:**
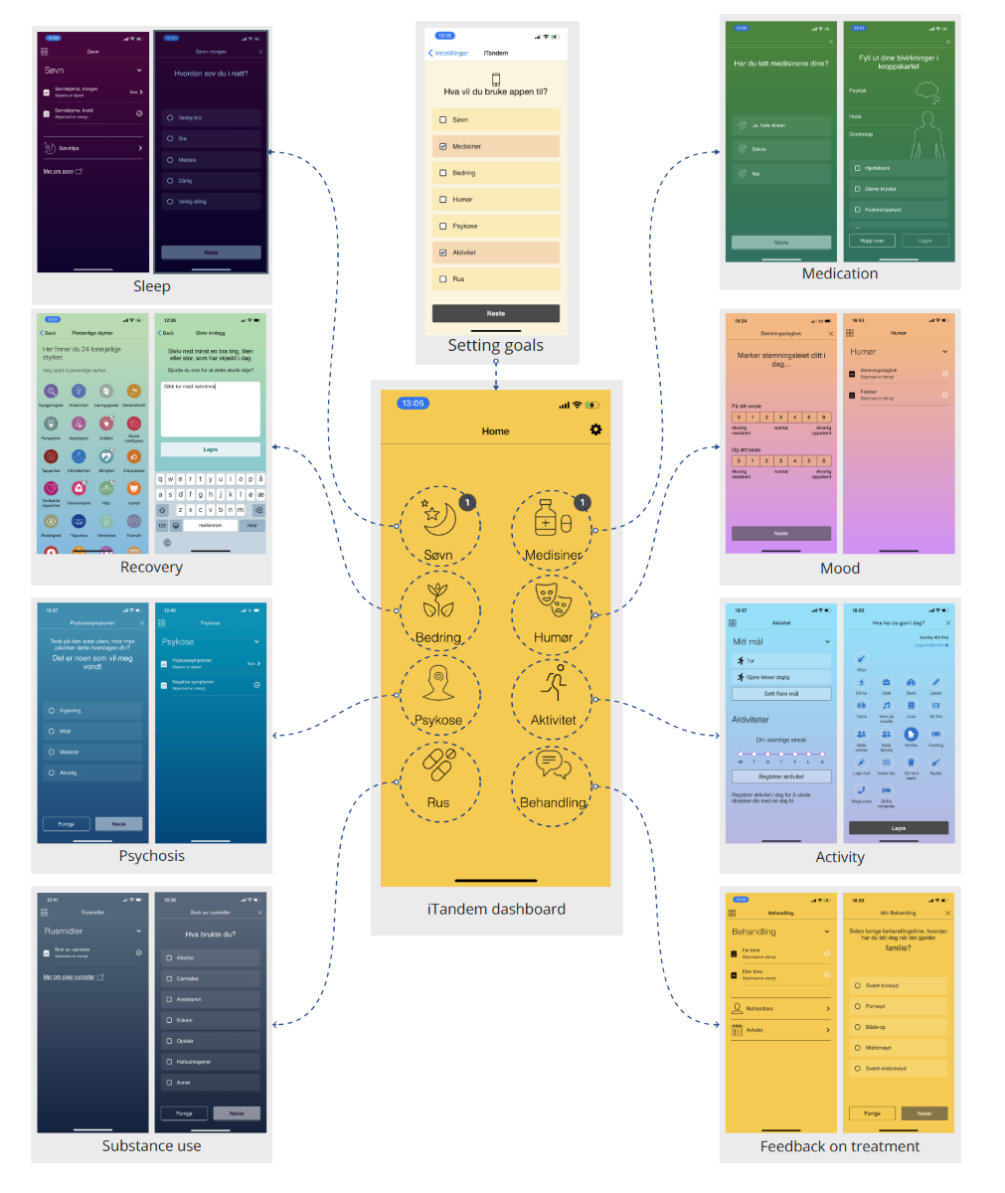
Screenshots illustrating the home page in iTandem and the page where you choose which modules you want to use (setting goals). The other pages show the various modules. Left: sleep, recovery, psychosis, and substance use. Right: medication, mood, activity, and feedback on treatment.

The final module of iTandem was included to adhere to the Norwegian guidelines for psychosis treatment, which underline the need for better tools to evaluate the experience of treatment. PROMs and patient-reported experience measures (PREMs) are widely used [68]. PROMs are primarily used for the assessment of treatment outcomes, while PREMs are used to evaluate patients’ experiences with the service they receive [69]. They allow for more direct feedback to the provider. In a tool designed for therapeutic collaboration and alliance building, the service users’ experience and ability to provide feedback on the sessions were considered important for iTandem. Feedback on treatment is also a fundamental principle in cognitive behavioral therapy, which is one of the most frequently used treatment approaches in mental health care. Therefore, including this module would also support cognitive behavioral therapy. The module consisted of questions about how the last week had been, and questions about the individual treatment sessions, to be answered before and after each session (Figure 1).

#### Report

**Figure 2:**
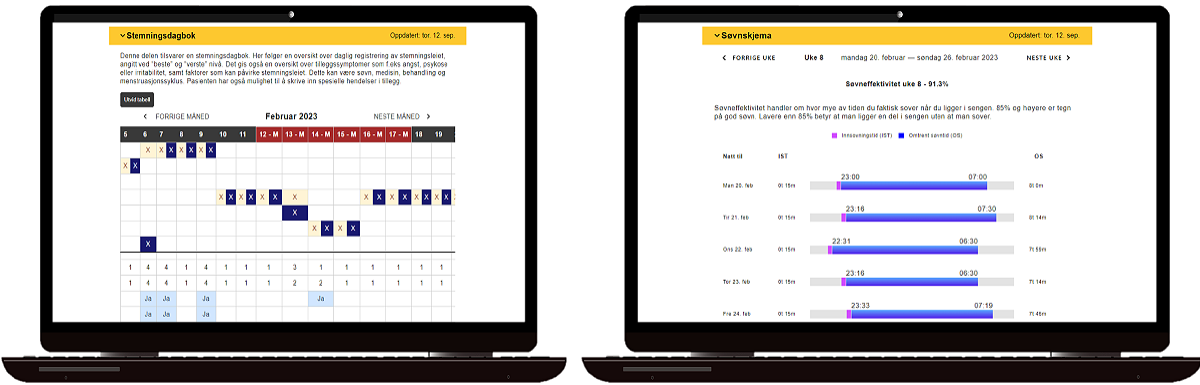
Illustration of 2 reports based on data logged into iTandem and how they will look on a screen. To the left is a mood diary illustrating data capturing the highest and the lowest mood during a day. To the right is a sleep diary, visualizing sleep during a week. The sleep period and sleep effectiveness are marked in different colors. A higher resolution version of this image is available as Multimedia Appendix 1.

Both clinicians and service users wanted a report to be able to log patterns. However, due to data security issues, only a limited amount of data could be stored on the phone. Except for the positive diary, activity, and medication list, data from all the modules were presented in the report. The positive diary was excluded from the report to allow service users to record positive events without feeling compelled to share them with clinicians. The Activity module incorporated elements of gamification, such as weekly streaks, which were believed to be a sufficient motivator. The other data entered were transferred to TSD. A report was available for clinicians, with the purpose of being shared with service users during sessions. Design principles were applied to make the report as easy to read as possible. Patients were not able to log into the report themselves. This decision was influenced by both technical and regulatory challenges, as well as how iTandem was intended to function as a tool for collaboration and shared decision-making. By integrating the report reading into the sessions, we believe it better supports the main goal (Figure 2).

### Beta Testing

#### Overview

**Figure 3:**
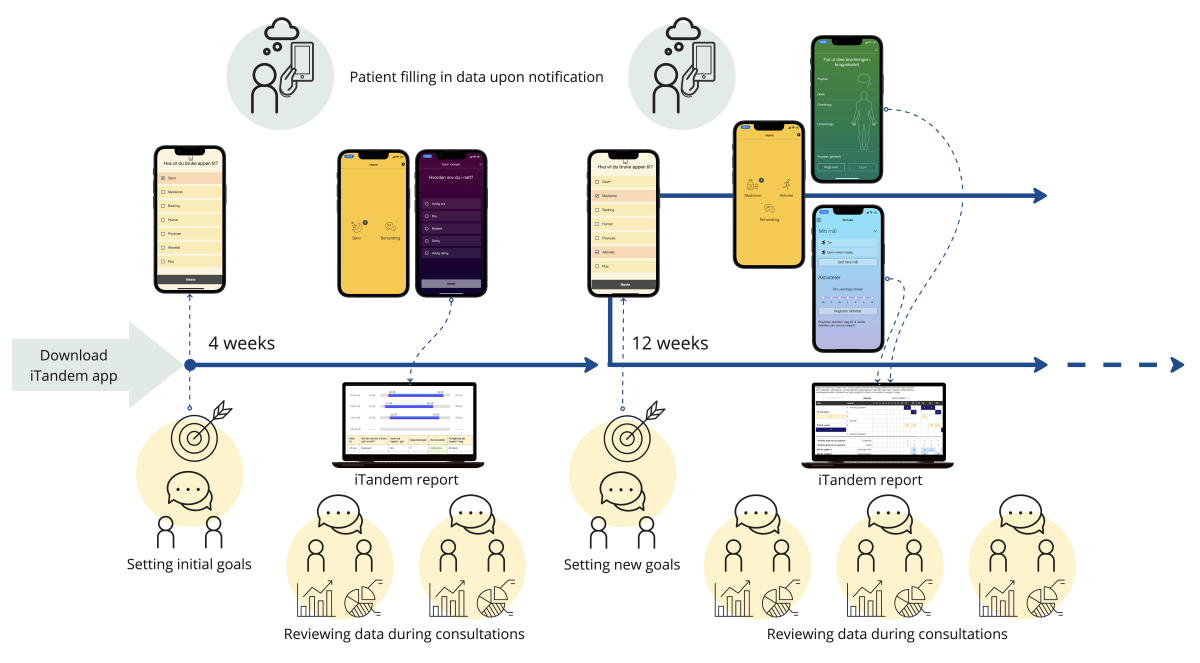
A workflow with iTandem. After setting initial goals, the service user logs data, which are assessed during sessions. After monitoring sleep for 4 weeks, the service user and the clinician choose to change modules as they want to focus more on activities and medication.

Six clinicians (2 psychologists and 4 psychiatrists) were invited to participate in beta testing and attended a 3-hour training session on how to use iTandem. During this session, we demonstrated the different modules and their intended purposes. In addition, we provided an online protocol where participating clinicians and service users could access written information about the content and how to use the app. In collaboration with clinicians, service users were encouraged to choose 2-3 modules relevant to their treatment. It was not emphasized that this was set as an upper limit, but both clinicians and designers were concerned about overwhelming the patients with too many notifications. This could also undermine the overall goal of helping to focus sessions on issues considered particularly relevant to the patient. The patients used the app for 6 weeks (Figure 3).

After beta testing, 4 service users and 5 clinicians agreed to be interviewed about their experiences with iTandem. This served as a preliminary proof of concept to determine whether the concept appeared to have potential. We also wanted feedback to adjust both iTandem and the educational implementation program before proceeding with a larger test in a clinical setting. Several issues were addressed, and the responses were sorted based on what appeared to be the most relevant attitudes and sentiments following the test.

#### Feedback on Concept by Service Users

The service users were mainly positive toward iTandem and the possibilities it offered. They appreciated the ability to select different modules that captured issues beyond the symptoms of their primary diagnosis, working alongside their clinician. This approach made it easier for them to focus on subjects and issues that were important to them and to convey this effectively to their clinician, thus supporting collaboration on goal setting and shared decision-making. They had concerns about how the report was used during sessions and emphasized the importance of clinicians utilizing the logged data; otherwise, the aspect of collaboration would be undermined. Some service users felt that the questions in the app were a bit repetitive (mainly the validated forms), and called for more opportunities to use free text to convey important information to their clinicians. It should be noted that there were service users who signed the informed consent to participate but did not follow through. According to clinicians, this was related to either paranoid ideas, high general symptom load, or being transferred to other departments.

#### Feedback on the Concept by Clinicians

Clinicians found the data from iTandem in the report to be useful; it provided important insights for their therapeutic work. Several were surprised by how the focus of treatment shifted when they received new information through the app, aligning with the goal of shared decision-making and increased collaboration. However, they found it was not always easy to remember to access and read the report, and they called for the report to be made accessible in the regular hospital records. By contrast, when this was not an option, they found the final solution to be manageable.

#### Implementation Issues

Many service users were eager to try several modules at once. However, notifications from multiple modules were perceived as overwhelming, highlighting the importance of working with 2-3 modules at the same time. The time spent on each module was deemed appropriate. Clinicians also felt overwhelmed by the challenge of integrating a digital tool into treatment sessions while finding space for it in their clinical routine. They found it stressful not to be fully familiar with the tool, making it difficult for them to effectively explain its use to the service user.

#### Technical Issues

Several technical issues arose regarding both the data sent from the app to TSD and the presentation in the report. This was partly due to errors in the app, but also because the report functionality was newly developed by the web app group at USIT and had not been properly tested beforehand. This led to several periods of downtime for the report. Even though this was seen as a major problem that persisted for several weeks, participants continued to enter data and found it meaningful. Furthermore, there were issues regarding the user-friendliness of the clock in the Sleep module. It was too easy to enter the wrong date, which obviously resulted in incorrect sleep patterns in the report. We also discovered missing data in the report.

#### Revision After Beta Testing and Plans for Pilot Testing

Based on the beta testing, we managed to resolve the technical issues. We revised the information material and teaching manual, omitting text and focusing on video films to show and tell how the app is intended to be used. In addition, we only recommend using a limited number of modules at a time to avoid overwhelming notifications. The new information material, manual, and the revised iTandem have since been further tested in a pilot trial conducted during winter 2023. In this trial, we tested iTandem as part of an early intervention service in a rural area in Norway. Data from this trial are currently being analyzed and will be part of a PhD thesis on how digitalization impacts service delivery in mental health care.

Furthermore, several studies are planned, incorporating iTandem as part of studies that use single modules and investigate implementation research connected to digitalization in mental health care.

Discussion

We have developed and beta tested a digital tool, iTandem, based on principles from design thinking. The main aim has been to utilize technology to strengthen and focus shared decision-making between service users and clinicians in mental health care and to enhance goal setting. The demand for simple functionality was underlined by both service users and clinicians.

We believe the design process was helpful in designing a tool that accommodates shared decision-making. We found the tool to follow many of the principles recommended by Priebe and colleagues [10], such as a heightened focus on the service users’ concerns, positive regard and personal respect, appropriate involvement of service users in decision-making, genuineness with personal touch, and the use of a psychological treatment model [10]. By allowing service users to choose modules to aid focus in treatment, we aim to empower them during their recovery process.

What is meaningful for the user should be the priority in treatment. This does not mean that clinical evaluation is less important, but to build an alliance and secure a therapeutic relationship, the service users’ interests must remain in focus throughout therapy. This aligns with the demand for more patient-centered care. It is also in line with the shift from “patient-centered” to “person-centered” care, as discussed by Boardman and Dave [70]. There is a change in how we view the therapeutic relationship, where the therapeutic alliance is more about co-production as a key element in the clinician-service user collaboration.

Furthermore, we believe that the ability to choose themes to work on and the possibility to give feedback on treatment sessions, including questions about being met with respect, will underpin service users’ experience of being respected and treated in a nonjudgmental way. We hope this tool will enhance clinicians’ motivation to make changes in accordance with the service users’ experienced needs. By using design both as a creative process and applied to the visual elements, we have strived to make the tool engaging and less “clinical.” This may increase the feeling of a more personal touch. To accommodate a more generic therapeutic use, the elements of iTandem are well suited to fit cognitive behavioral therapy, focusing on agenda setting, goal setting, and the execution of activities. Unsurprisingly, a design process led to solutions that align well with this therapy approach, as it is collaborative, pragmatic, structured, and focused on current problems.

We have previously worked with Boundary Objects Theory, where using a digital object can be seen as a way to transfer knowledge between different knowledge domains [71]. Based on this work, for an object to function effectively, it needs to inherit features such as dynamism, flexibility, standardization, and a shared structure [72]. We believe iTandem has the potential to work as a boundary object. It accommodates the needs of the service user, and it is simple to engage in, hence adhering to the KISS principle. iTandem allows for the transfer of knowledge between service user and clinician with an inherent flexibility yet standardized approach. The ability to collaborate via this object allows for a deeper understanding of the problem, leading to more focused problem-solving.

iTandem differs from the numerous assessment tools offered as PROMs in Norwegian mental health care services at the moment. The positive aspects of PROMs lie in their basis on validated measures that aim to track progress in therapy. However, the extensive number of questions to be answered can be overwhelming, and the amount of data may be challenging for clinicians to manage and oversee effectively. PROMs often cover broad categories, and their questions can sometimes seem less relevant to the service users’ specific problems. Therefore, it may be necessary to scale down the assessments to focus on a minimum that allows for qualified evaluations of how treatment progresses. This approach can alleviate the burden on clinicians and service users by combining different tools, such as apps like iTandem and more focused classic PROMs. Utilizing language and questions that are closely aligned with a clinical setting might be sufficient to establish a rapport and gather relevant information about progress.

Another important aspect is how we provide flexibility in services. While we offer options such as video consultations, PROMs, apps, and similar tools, the solutions presented to service users often rely on traditional approaches, as if one size fits all. Alternating between different tools and formats during treatment sessions could, in our view, make the treatment more effective. Insights from user reviews of mental health apps indicate that highly rated apps tend to offer a variety of options, functionalities, and content for users to choose from [73]. This is particularly relevant to attrition rates, as different tools may be needed during various phases of the recovery process. A lack of use could even be a positive sign, indicating that the problem has been resolved and the user has moved on. Additionally, Alqahtani et al [73] identified several common weaknesses of mental health apps, including poor usability, lack of personalization, credibility issues, security concerns, and inadequate customer service. We believe these findings align with the need for greater flexibility in services.

To address the future needs of health care services, we believe it is essential to examine how digital tools can be integrated into regular workflows. Greater insight is needed into the barriers and facilitators of implementation, as well as how health technology assessment methods can be used to evaluate which technologies benefit service users, clinicians, and organizations while also being economically effective.

## Data Availability

Because of the small sample size and limitations in the written informed consent, the data are not available to other researchers.

## Appendix

Multimedia Appendix 1 Higher Resolution version of Figure 2.

## Abbreviations

KISS: keep it simple, stupid
OUH: Oslo University Hospital
PREM: patient-reported experience measure
PROM: patient-reported outcome measure
TIPS SE: Early Intervention in Psychosis Advisory Unit for South East Norway
TSD: Services for Sensitive Data Tjenester for Sensitive Data
UiO: University of Oslo
USIT: University of Oslo’s Center for Information Technology
UX: user experience
