# Supplementary figures and images for: A Mobile Health Intervention to Support Collaborative Decision-Making in Mental Health Care: Development and Usability
Source: JMIR Form Res. 2025 Jan 17;9:e57614. doi: 10.2196/57614 (PMC11786142; doi:10.2196/57614)

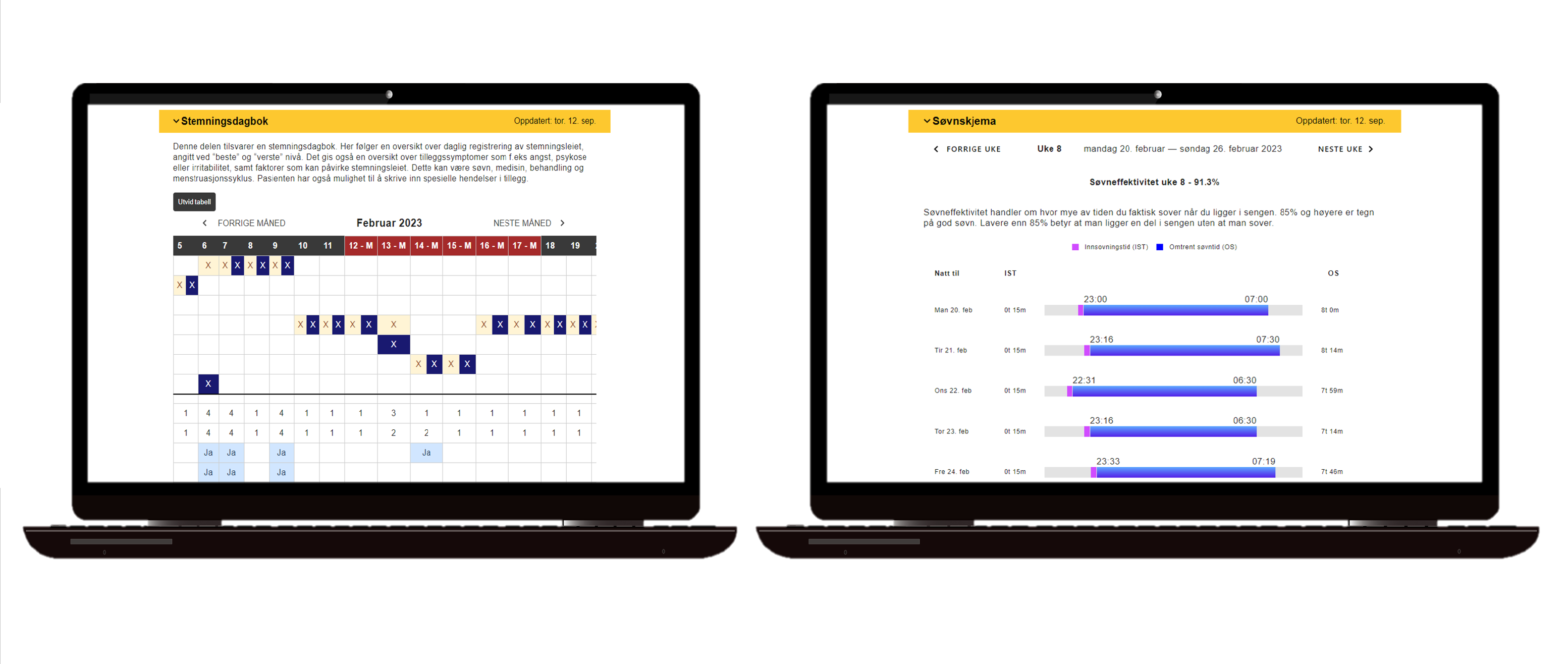

Supplement: Multimedia Appendix 1 [file formative_v9i1e57614_app1.png]
